# Anandamide inhibits breast tumor-induced angiogenesis

**Published:** 2014-04-08

**Authors:** P Picardi, E Ciaglia, MC Proto, S Pisanti

**Affiliations:** 1 Department of Medicine and Surgery, University of Salerno, Via Salvatore Allende, Baronissi (SA), Italy; 2 Department of Pharmacy, University of Salerno, Via Giovanni Paolo II, Fisciano (SA), Italy (spisanti@unisa.it)

**Keywords:** angiogenesis, endocannabinoid system, breast cancer, anandamide

## Abstract

Breast cancer is one of the most frequently diagnosed malignancies and a leading cause of cancer death in women. Great advances in the treatment of primary tumors have led to a significant increment in the overall survival rates, however recurrence and metastatic disease, the underlying cause of death, are still a medical challenge. Breast cancer is highly dependent on neovascularization to progress. In the last years several anti-angiogenic drugs have been developed and administered to patients in combination with chemotherapeutic drugs.

Collected preclinical evidence has proposed the endocannabinoid system as a potential target in cancer. The endocannabinoid anandamide has been reported to affect breast cancer growth at multiple levels, by inhibiting proliferation, migration and invasiveness in vitro and in vivo and by directly inhibiting angiogenesis. Aim of the present work is to investigate if anandamide is able to affect the proangiogenic phenotype of the highly invasive and metastatic breast cancer cells MDA-MB-231. We found that following anandamide treatment, MDAMB-231 cells lose their ability to stimulate endothelial cells proliferation in vitro, due to a significant inhibition of all the pro-angiogenic factors produced by these cells. This finding adds another piece of evidence to the anti-tumor efficacy of anandamide in breast cancer.

## I. INTRODUCTION

Breast cancer is the most common malignancy in women, excluding skin cancer, and is the second for mortality following lung cancer [1]. Great improvements in a comprehensive treatment for breast cancer have led to a significant increment in the overall survival rates, however recurrence and metastatic disease still represent an urgent medical problem. A huge amount of studies have demonstrated that breast cancer, like many solid tumors, depends on vascular growth to progress. Indeed, breast tumor angiogenesis is considered as an independent prognostic factor for breast cancer [2]. Several currently used conventional therapeutic regimens, such as numerous chemotherapeutic agents and endocrine therapies, show an intrinsic anti-angiogenic efficacy. Moreover, specific drugs that target angiogenesis have been developed. Among these, the most famous is the VEGF-neutralizing monoclonal antibody bevacizumab [3]. Unfortunately, numerous clinical trials conducted in breast cancer have revealed that this malignancy is modestly sensitive to bevacizumab, even when combined with standard chemotherapy protocols, showing a lack of survival advantage. These disappointing results led to recently discontinue its FDA approval in breast cancer treatment [4].

The endocannabinoid system, whose complex biological regulatory functions have been disclosed as crucial especially in several patho-physiological conditions, is emerging as a potential new target in cancer, due to the numerous evidence collected also in preclinical studies [5]. Natural and synthetic cannabinoids and compounds belonging to the endocannabinoid system (lipid molecules containing long-chain polyunsaturated fatty acids, amides, esters and ethers, like anandamide and 2-arachidonoylglycerol (2-AG)), have been reported to affect tumor growth at more than one step in several types of malignancies. Indeed, they have been shown to inhibit cell proliferation and induce cell death, mainly through apoptosis and autophagy, but also to prevent tumor progression, metastasis formation and to inhibit oxygen and nutrient supply through a direct effect on tumor neovascularization [5]. As regard to breast cancer, CB1 and CB2 receptors expression was found in several breast cancer cell lines with peculiar oncogenic patterns and different metastatic potential, as well as in human breast tumor tissues [6,7]. Cannabinoids and endocannabinoids-related compounds were able to affect breast cancer cell proliferation, inducing apoptosis, and to inhibit migration and invasiveness [8–11]. The metabolically stable analogue of anandamide, Met-F-AEA, was reported to inhibit the proliferation of the estrogen receptor negative (ER-) MDA-MB-231 breast cancer cells, inducing a S phase cell cycle arrest correlated with DNA damage and Chk1 activation [12,13]. Anandamide inhibited also HMG-CoA reductase activity, thus affecting the pattern of expression of oncogenic prenylated proteins involved in the proliferation and metastatic potential of breast cancer cells, such as Ras and RhoA [14]. Indeed, anandamide was able to reduce the invasiveness of highly metastatic MDA-MB-231 cells, inhibiting their migration through the RhoA signalling pathway [12, 15]. The efficacy of anandamide was maintained in the *in vivo* setting, since it was able to reduce the number and dimension of metastatic nodes in a mouse model of metastatic spreading [12]. Interestingly, we previously demonstrated that anandamide also directly affects angiogenesis, inhibiting endothelial cell proliferation, tube formation and neovascularization *in vivo* [16]. Aim of this study is to investigate if anandamide is able to affect the proangiogenic phenotype of breast cancer cells, adding another piece of evidence to its anti-tumor efficacy.

## II. METHODOLOGY

### Cell lines

The human breast cancer cell line MDA-MB-231was from Interlab cell line collection (IST, Genova) and was grown in Dulbecco’s Modified Eagle Medium (DMEM) supplemented with 10% fetal bovine serum. Human umbilical endothelial cells (HUVEC) were isolated from umbilical cords, as described [17] and grown in M199 medium supplemented with 10% fetal bovine serum, aFGF, bFGF, EGF, hydrocortisone and heparin. Cells were cultured at 37°C in a humidified 5% CO_2_ atmosphere.

### Drugs

Met-F-AEA (2-methyl-2’-F-anandamide), a metabolically stable analogue of anandamide, was purchased from Sigma-Aldrich. Met-F-AEA is indicated in the text as anandamide (AEA).

### Proliferation assay

Cell proliferation was evaluated, *in vitro*, by measuring [^3^H]-thymidine incorporation. In brief, 5×10^3^ cells/ml were seeded into 96-well plates, immediately treated with the drugs, incubated for 24 h at 37°C (5% CO_2_), then pulsed with 0.5 μCi/well of [^3^H]-thymidine (Amersham Biosciences Europe, Italy) and harvested 4 h later. Radioactivity was measured in a scintillation counter (Wallac, Turku, Finland).

### Human angiogenesis array

Human angiogenesis antibody array on MDA-MB-231 tumor-conditioned medium was performed following manufacturer’s instructions (RayBiotech, Inc.). Culture supernatants corresponding to the final 24 h of incubation with anandamide (10 µM) were collected and assayed for angiogenic factors production. The array membranes were incubated with tumor-conditioned medium, stained using a chemiluminescence system (ECL-Amersham Biosciences) and then exposed to X-ray films (Kodak). Immunoreactive spots were quantified using Quantity One 1-D analysis software (Bio-Rad).

### Statistical analysis

Statistical computations were done using the GraphPad prism 6.0 software (San Diego, CA, USA). Data obtained from multiple experiments were calculated as mean±SD and analysed for statistical significance using Student’s T test or 1-way ANOVA for independent groups, with Tukey correction for multiple comparisons. P values less than 0.05 were considered significant.

## III. RESULTS

**Fig. 1.**
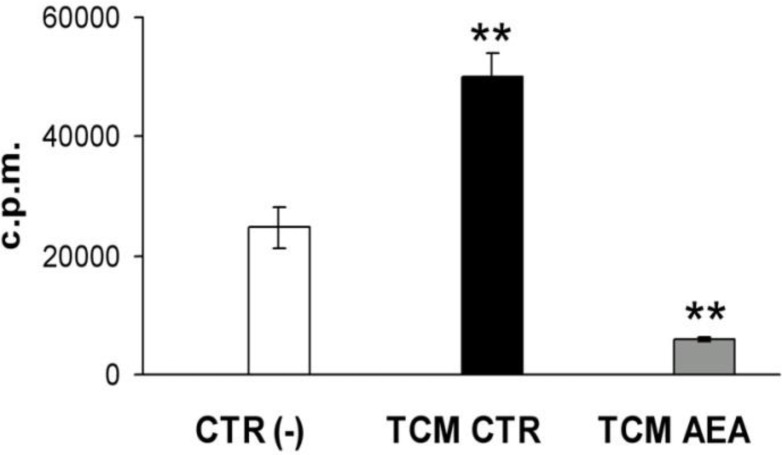
**Tumor conditioned medium from anandamide-treated MDAMB-231 inhibits angiogenesis.** The graph reports the [^3^H]-thymidine incorporation levels (counts per minute) in endothelial cells expressed as mean ± SD values of three experiments in triplicates. ANOVA, ^**^P<0.01. CTR, control; AEA, anandamide.

In order to evaluate the effect of anandamide on breast cancer-induced angiogenesis, we performed a simple *in vitro* angiogenesis assay using as angiogenic stimulus the tumor-conditioned medium (TCM) derived from MDAMB-231 cells. Briefly, MDA-MB-231 cells were treated with anandamide (10 μM, 24 h) and then the TCM was collected and tested on human umbilical vein endothelial cells (HUVEC) proliferation by [^3^H]-thymidine incorporation into DNA. As expected, TCM significantly induced endothelial basal proliferation *vs* control. Interestingly, we found that in the presence of TCM from anandamide-treated MDA-MB-231 cells, the proliferation of endothelial cells was strongly reduced (Fig. 1).

This could be the result of an anandamide-switched balance between pro-angiogenic and anti-angiogenic factors produced by breast cancer cells in behalf of angiogenesis inhibitors. The next step was indeed to investigate the nature and levels of angiogenic molecules present in breast cancer TCM and their modulation after anandamide treatment. To this end we used an angiogenesis protein array. We found that, for all tested pro-angiogenic factors, there was a reduction trend following anandamide treatment (Table 1).

In particular, IFNγ, leptin, TGFβ1, TIMP1, TIMP2, thrombopoietin and VEGF levels were significantly inhibited by anandamide in the conditioned medium of MDA-MB-231 breast cancer cells.

## IV. DISCUSSION

In the present work we demonstrated for the first time that a metabolically stable anandamide analogue, Met-F-AEA, already known to have an interesting anti-tumor efficacy in several tumor types including breast cancer [12–15], along with a direct anti-angiogenic activity on endothelial cells [16], was able to affect the pro-angiogenic phenotype of the highly metastatic MDA-MB-231 breast cancer cells. Indeed, following anandamide treatment, MDAMB-231 cells lost the capability to stimulate endothelial cells proliferation *in vitro*, due to a significant inhibition of all the pro-angiogenic factors produced by tumor cells and usually responsible of their highly pro-angiogenic phenotype, allowing tumor cells to get nourish and thus to growth [18].

Our data (Fig. 1 and Table 1) show that the conditioned medium from breast cancer cells contains an amount of potent angiogenic factors and pro-inflammatory cytokines, as detected by an angiogenesis array.

Among the factors secreted in large amounts in the tumor conditioned medium and deeply inhibited after treatment with anandamide in comparison to the control, there is VEGF (47% of inhibition *vs* control). VEGF is one of the major promoters of angiogenesis, through the binding to its tyrosine kinase receptors EGFR-1/Flt-1 and VEGFR-2/KDR/Flk-1 it is able to regulate more than one step in angiogenesis, from endothelial cell survival and proliferation, to migration and vascular permeability [19]. As a critical factor in the induction of angiogenesis, VEGF has become an attractive target for the development of anti-angiogenic drugs, the most famous of whose is the VEGF-neutralizing monoclonal antibody bevacizumab [3]. Several solid cancers, such as colon and lung cancer, have been successfully treated with bevacizumab, whereas its approval in breast cancer has been recently revoked [20]. Indeed, despite the success of bevacizumab and other anti-angiogenic drugs, it has to be taken into account that even in responsive patients, anti-angiogenic drugs generally prolong survival only in the order of few months and the clinical outcome is associated with the development of resistance and an increased risk of invasion and metastasis [2]. This could be due to the high redundancy of angiogenic molecules that tumors can produce to provide oxygen and nutrients stimulating their own growth and vascularization. Moreover, tumors can also switch between different types of vascularization that don’t necessarily require VEGF and its signalling. Therefore, strategies aimed to target angiogenesis at multiple levels could improve the efficacy and reduce the risk of resistance development. Interestingly, we found that anandamide is able to inhibit multiple angiogenic molecules beyond VEGF, thus significantly affecting the pro-angiogenic phenotype of breast cancer cells.

In our study, we reported a significant reduction in the levels of the adipocytokine leptin, that has been reported to stimulate mammary tumor growth. Furthermore high levels of leptin in women correlate to an increased risk of breast cancer, metastatic tumor phenotype and poor prognosis [21,22]. Leptin stimulates angiogenesis and breast cancer invasiveness through the enhanced neovascularization, so it is a key factor to control breast cancer progression [23].

Noteworthy, most of the other growth factors and chemokines inhibited by anandamide treatment (IL8, GRO, ENA-78) have been reported to play a significant role in mediating neovascularization during tumorigenesis in several tumor types and have been shown to be of great importance in tumor progression [18]. These chemokines are under the regulation of PPARγ pathway and PPARγ ligands have been shown to inhibit tumor-associated angiogenesis by blocking the production of ELR+CXC chemokines [24]. It has been reported that anandamide induces transcriptional activation of PPARγ, binding directly to PPAR-ligand binding domain [25,26]. Therefore this could be the mechanism by which anandamide is able to inhibit the production of these angiogenesis-stimulating molecules, thus inhibiting breast cancer neovascularization.

## V. CONCLUSION

In conclusion, our study adds another piece of evidence to the anti-tumor efficacy of the endocannabinoid anandamide in breast cancer.

## Figures and Tables

**TABLE 1.** ANGIOGENIC FACTORS SECRETED BY MDA-MB-231 BREAST CANCER CELLS FOLLOWING ANANDAMIDE TREATMENT

**Angiogenic factors**	**TCM**	**TCM+ AEA**	**%**
Angiogenin	20 ± 0.8	19.3 ± 2.3	5%
ENA-78	3.8 ± 1.4	2.7 ± 1.5	29%
bFGF	10.2 ± 2	9.8 ± 3.3	4%
GRO	12.5 ± 0.1	10.7 ± 1.5	15%
IFNγ	1.4 ± 0.07	1.1 ± 0.08^**^	21%
IL6	18 ± 0.5	14.9 ± 4.6	17%
Leptin	2.2 ± 0.2	0.9±0.07^***^	58%
MCP-1	57.1 ± 6.0	49.4 ± 9.5	13%
PDGF-BB	9.7 ± 1.9	6.8 ± 1.1	29%
PlGF	10.5 ± 1.8	8.3 ± 1.2	21%
RANTES	8.6 ± 0.1	7.9 ± 0.5	7%
TGFβ1	35.2 ± 3.7	26.7 ± 1.8^*^	23%
TIMP1	37.1 ± 2.6	30.2 ± 1.8^*^	18%
TIMP2	75.7 ± 10.7	50 ± 2.8^*^	33%
Thrombopoietin	10.5 ± 1.3	6.8 ± 0.6^*^	35%
VEGF	42.6 ± 7.6	22.4 ± 3.2^*^	47%
VEGF-D	10.6 ± 2.3	6.8 ± 1.9	36%

Data are presented as mean ± SD of three experiments. % = percentage of inhibition *vs* TCM.,

* P <0.05,

** *P* < 0.01,

*** *P* < 0.001; Student’s T test.
